# Interaction of Nuclear Export Protein with G Protein Pathway Suppressor 2 (GPS2) Facilitates Influenza A Virus Replication by Weakening the Inhibition of GPS2 to RNA Synthesis and Ribonucleoprotein Assembly

**DOI:** 10.1128/JVI.00008-21

**Published:** 2021-04-26

**Authors:** Wenxiao Gong, Xinglin He, Kun Huang, Yufei Zhang, Chengfei Li, Ying Yang, Zhong Zou, Meilin Jin

**Affiliations:** a State Key Laboratory of Agricultural Microbiology, Huazhong Agricultural University, Wuhan, People's Republic of China; b College of Veterinary Medicine, Huazhong Agricultural University, Wuhan, People's Republic of China; c College of Life Sciences, Huazhong Agricultural University, Wuhan, People's Republic of China; d Key Laboratory of Development of Veterinary Diagnostic Products, Ministry of Agriculture, Wuhan, People's Republic of China; Hudson Institute of Medical Research

**Keywords:** GPS2, NEP, influenza, virus-host interactions, vRNP assembly

## Abstract

The nuclear export protein (NEP) serves multiple functions in the life cycle of influenza A virus (IAV). Identifying novel host proteins that interact with NEP and understanding their functions in IAV replication are of great interest. In this study, we screened and confirmed the direct interaction of G protein pathway suppressor 2 (GPS2) with NEP through a yeast two-hybrid screening assay and glutathione *S*-transferase pulldown and coimmunoprecipitation assays. Knockdown or knockout of GPS2 enhanced IAV titers, whereas overexpression of GPS2 impaired IAV replication, demonstrating that GPS2 acted as a negative host factor in IAV replication. Meanwhile, GPS2 inhibited viral RNA synthesis by reducing the assembly of IAV polymerase. Interestingly, IAV NEP interacted with GPS2 and mediated its nuclear export, thereby activating the degradation of GPS2. Thus, NEP-GPS2 interaction weakened the inhibition of GPS2 to viral polymerase activity and benefited virus replication. Overall, this study identified the novel NEP-binding host partner GPS2 as a critical host factor to participate in IAV replication. These findings provided novel insights into the interactions between IAV and host cells, revealing a new function for GPS2 during IAV replication.

**IMPORTANCE** NEP is proposed to play multiple biologically important roles in the life cycle of IAV, which largely relies on host factors by interaction. Our study demonstrated that GPS2 could reduce the interaction between polymerase basic 1 (PB1) and PB2 and interfere with viral ribonucleoprotein (vRNP) assembly. Thus, GPS2 inhibited the RNA synthesis of IAV and negatively regulated its replication. Importantly, IAV NEP interacted with GPS2 and mediated the nuclear export of GPS2, thereby activating the degradation of GPS2. Thus, NEP-GPS2 interaction weakened the inhibition of GPS2 to viral polymerase activity and benefited virus replication.

## Abstract (precis)

NEP is proposed to play multiple biologically important roles in the life cycle of IAV, which largely relies on host factors by interaction. Our study demonstrated that GPS2 could reduce the interaction between polymerase basic 1 (PB1) and PB2 and interfere with viral ribonucleoprotein (vRNP) assembly.

## INTRODUCTION

Influenza A virus (IAV) is a serious public health concern that causes annual seasonal epidemics and even sporadic pandemics, leading to severe morbidity and mortality worldwide (1, 2). IAV is responsible for approximately 290,000 to 650,000 deaths each year worldwide (http://www.who.int/topics/influenza/en/), and young children and elderly individuals are highly vulnerable to infection. IAV is an obligate intracellular parasite that heavily relies on the host cellular machinery to support its replication throughout its life cycle, from entry to budding. Therefore, identifying novel host factors that interplay with IAV protein and understanding the underlying molecular mechanism in IAV infection are crucial to controlling IAV replication. Recently, several large-scale screenings have been performed to identify new host proteins involved in IAV replication (3–5). Nevertheless, these host factors have not yet been completely identified.

IAV belongs to the *Orthomyxoviridae* family, and its genome comprises eight negative-sense single-stranded RNA segments encoding at least 13 viral proteins (6). Some of them have been recently identified, such as polymerase basic 1 (PB1)-F2, PB1-N40, and polymerase acidic protein (PA)-X (7–9). The nuclear export protein (NEP) is derived from alternatively spliced RNAs that are transcribed from RNA segment 8 of the viral genome (10) and is proposed to play multiple biologically important roles during the life cycle of IAV (11). NEP acts as an adaptor to mediate the export of viral ribonucleoproteins (vRNPs) from the nucleus to the cytoplasm through signal export in late-stage infection (12, 13). NEP also plays a critical role in overcoming the species barrier (14). Recently, our group has provided a novel mechanism for IAV to antagonize the innate immune response via NEP (15). Moreover, NEP has several important functions, such as modulating the accumulation of viral RNA during transcription and translation (11, 16, 17), as well as interacting with host proteins such as ATPase to benefit viral budding and with several nucleoporins to enable nucleocytoplasmic switch (18, 19). However, other functions of NEP warrant further study.

G protein pathway suppressor 2 (GPS2) is a ubiquitous protein originally identified as a G protein suppressor that interferes with JNK1 activation in the RAS/MAPK pathway in yeast (20). An increasing number of reports have confirmed the roles of GPS2 in transcriptional regulation, including transcriptional repression and activation (21–23). Previous studies have found that GPS2 is a stable component of the SMRT corepressor complex, which is involved in inhibiting cellular and viral transcription (24, 25). Downregulation of GPS2 abrogates SMRT-mediated repression activity, whereas its upregulation potentiates this activity (26–28). Aside from its transcriptional roles, GPS2 also participates in adipogenesis and inflammatory responses (29–31). However, the role of GPS2 in the IAV life cycle remains largely unknown.

Studies of our laboratory group and others on IAV NEP have demonstrated that it potentially interacts with host factors, including GPS2 (5, 32). In the present study, an in-depth functional analysis of GPS2 was performed. First, we confirmed GPS2 as an IAV NEP-interacting partner during IAV infection, and GPS2 served as a negative regulator to IAV by inhibiting polymerase assembly, RNA-dependent RNA polymerase (RdRp)-dependent viral RNA synthesis, and virus replication. We also demonstrated that NEP-GPS2 interaction was a positive regulator of IAV by mediating the nuclear export of GPS2 and promoting the degradation of GPS2 to alleviate the inhibition to polymerase activity and vRNP assembly. These findings detailed a new interaction and revealed a new function for GPS2 during IAV infection.

## RESULTS

### NEP interacted with GPS2 *in vitro*.

**FIG 1:**
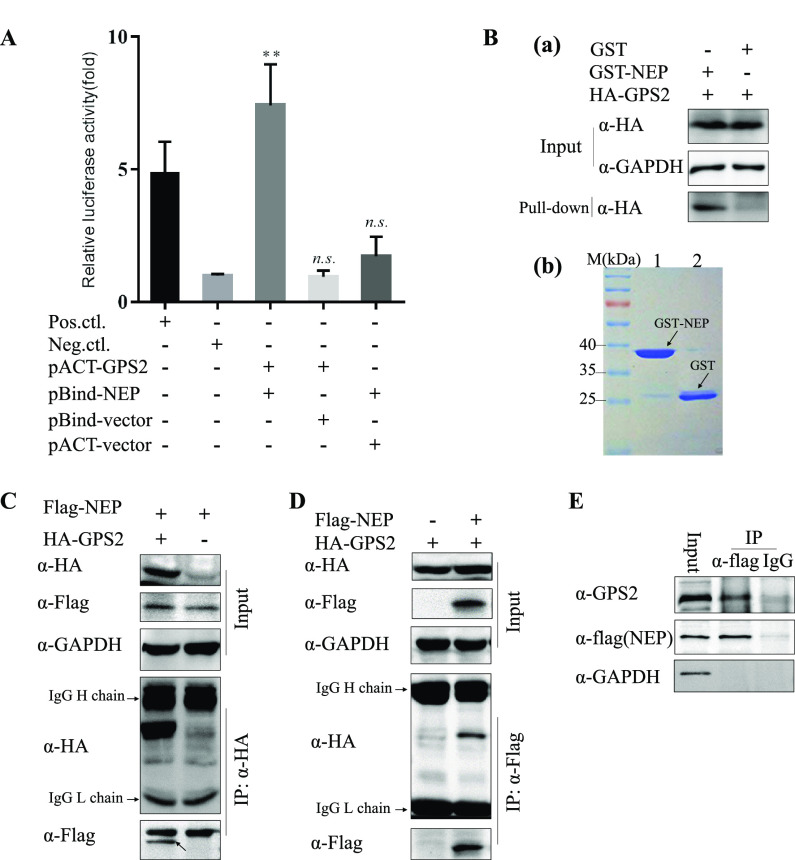
NEP interacted with GPS2. (A) NEP bound GPS2 in the MTH assay. HEK293T cells were cotransfected with pACT-GPS2, pBIND-NEP, and pG5luc, and then cell lysates were subjected to luciferase activity assays via MTH 24 h later. pBIND-ID and pACT-MyoD served as positive controls, and pACT and pBIND served as negative controls. The results are presented as the mean ± SD of three independent experiments (*, *P* < 0.05; **, *P* < 0.01; n.s., not significant; all by two-tailed Student's *t* test). (B) GST pulldown assay showing the direct interaction between NEP and GPS2. Lysates of HEK293T cells transfected with HA-GPS2 were incubated with an equal amount of GST or GST-NEP bound to glutathione-Sepharose 4B beads (b). Bound proteins were analyzed by Western blotting with an anti-HA monoclonal antibody (a). (C and D) Coimmunoprecipitation of HA-GPS2 and Flag-NEP in HEK293T cells. HEK293T cells were cotransfected with HA-GPS2 and Flag-NEP. Cell lysates were immunoprecipitated (IP) and immunoblotted (IB) with specific antibodies as indicated. (E) Coimmunoprecipitation of Flag-NEP in A549 cells. A549 cells were infected with WD-Flag-NEP virus at an MOI of 0.01, and cells were lysed 24 h postinfection. Cell lysates were IP and IB with specific antibodies as indicated.

A yeast two-hybrid screen of a human fetal brain cDNA library was performed to identify the host proteins that interact with IAV NEP (32). Nucleotide sequencing revealed that one of these positive clones was GPS2. Interaction between NEP and GPS2 was confirmed by cotransfection of plasmids pACT-GPS2 and pBind-NEP into HEK293T cells. A reporter gene assay was used to measure the differences in the relative strength of binding between two proteins. As shown in Fig. 1A, NEP interacted with GPS2 in 293T cells in the mammalian two-hybrid (MTH) system.

Glutathione *S*-transferase (GST) pulldown assays were performed using GST-NEP (expressed in Escherichia coli) and hemagglutinin (HA)-GPS2 fusion proteins (expressed in HEK293T) to further verify the interaction between NEP and GPS2. As shown in Fig. 1B, GST-NEP can directly interact with HA-GPS2 *in vitro*. Flag-NEP- and HA-tagged GPS2 or HA (negative control) were coexpressed in HEK293T cells, and a coimmunoprecipitation (co-IP) assay was performed using an anti-HA tag monoclonal antibody. Results showed that Flag-NEP was coprecipitated by HA-GPS2 (Fig. 1C). HA-GPS2 could be coprecipitated by Flag-NEP in a co-IP assay by using an anti-Flag tag monoclonal antibody, suggesting the direct binding of NEP to GPS2 (Fig. 1D). We then determined whether endogenous GPS2 could interact with NEP in IAV-infected cells. Co-IP results indicated that GPS2 could be coimmunoprecipitated in WD-Flag-NEP virus-infected A549 cell extracts when using a precipitation antibody to Flag tag, whereas GPS2 was undetected in the immunoprecipitates by a control IgG (Fig. 1E). Collectively, these data clearly demonstrated that NEP can physically bind to GPS2.

### GPS2 modulated IAV replication in cultured cells.

**FIG 2:**
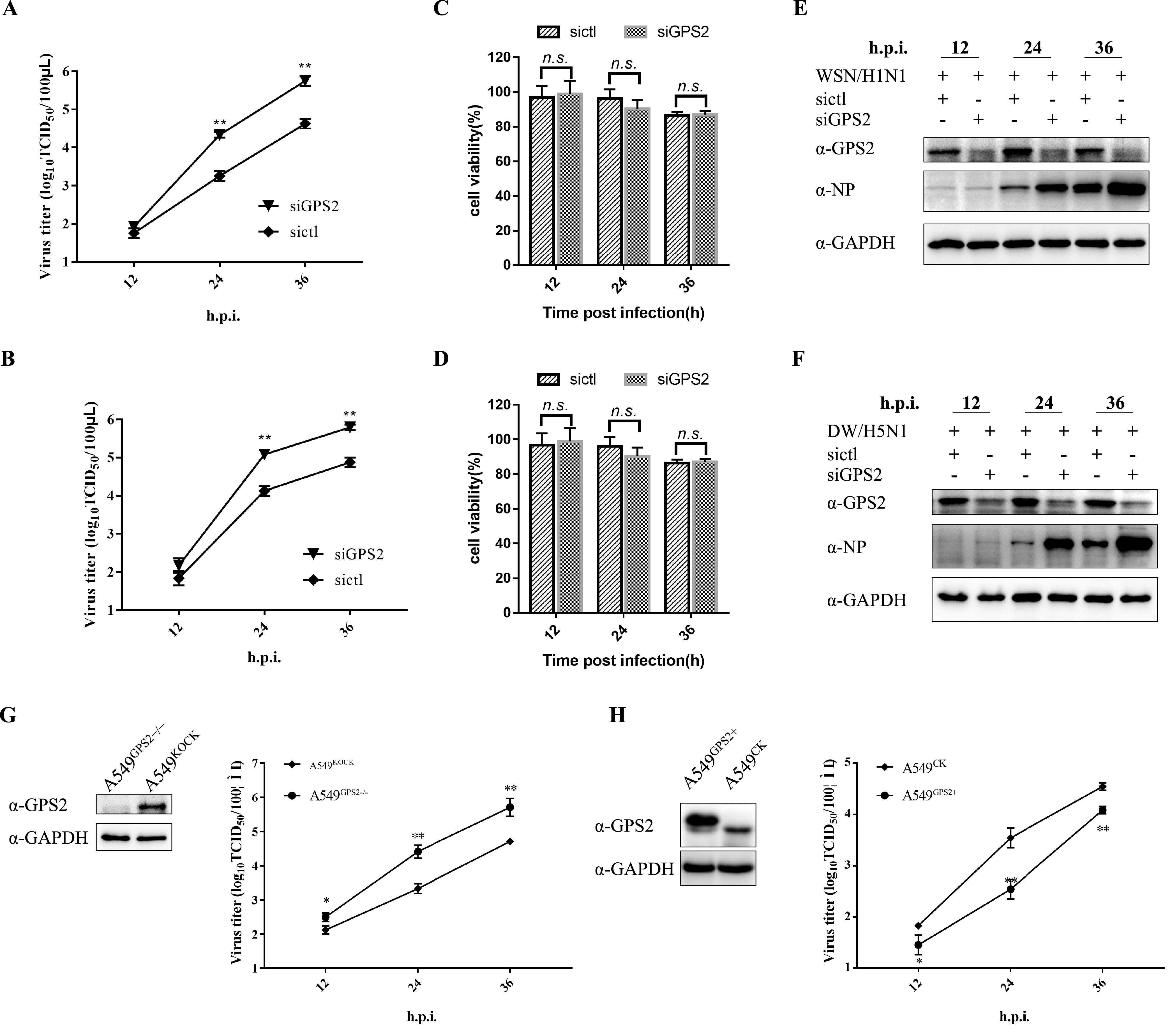
Effect of GPS2 on IAV replication. (A and B) A549 cells were transfected with sictl or siGPS2 for 24 h, followed by infection with the A/WSN/33 (WSN/H1N1) (A) or A/chicken/Hubei/327/2004 (DW/H5N1) (B) virus at an MOI of 0.01. Supernatants of the cell culture were collected at different times postinfection as indicated and assayed for virus titers by TCID_50_. (C and D) Effect of siGPS2 on A549 cell viability. A549 cells were transfected with siGPS2 or negative-control sictl. Cell viability was measured by cell counting kit 8 (CCK-8) assay at the indicated time points posttransfection. (E and F) Whole-cell lysates were assayed by Western blotting with the indicated antibodies. (G) GPS2-KO A549 cells (A549^GPS2−/−^) or empty retrovirus-transduced control A549 cells (A549^KOCK^) infected with the A/WSN/33 (WSN/H1N1) at an MOI of 0.01. Supernatants of the cell culture were collected at different times postinfection as indicated and assayed for virus titers by TCID_50_. (H) HA-GPS2 stable-expressed A549 cells (A549^GPS2+^) or empty retrovirus-transduced control A549 cells (A549^CK^) infected with A/WSN/33(WSN/H1N1) at an MOI of 0.01. Supernatants of the cell culture were collected at different times postinfection as indicated and assayed for virus titers by TCID_50_. For all experiments, results are presented as the mean ± SD from three independent experiments (*, *P* < 0.05; **, *P* < 0.01; n.s., not significant; all by two-tailed Student's *t* test).

Subsequently, we determined whether GPS2 affected IAV replication. To this end, specific small interfering RNA (siRNA) targeting GPS2 was used to knock down GPS2 in A549 cells, whereas siRNA served as a negative control (sictl). The silencing efficiency of GPS2-targeting siRNA was examined by Western blotting. Results showed that GPS2 expression was significantly downregulated by GPS2-targeting siRNA but unaffected by sictl (Fig. 2E and F). Then, we infected siRNA-treated cells grown in 24-well plates with IAV at a multiplicity of infection (MOI) of 0.01 to evaluate the effect of GPS2 knockdown on IAV replication. The supernatants were collected, and virus titers were measured by 50% tissue culture infective dose (TCID_50_). As shown in Fig. 2A and B, the viral titers of A/WSN/33 (WSN H1N1) and A/chicken/Hubei/327/2004 (DW H5N1) viruses were significantly enhanced in the GPS2-silenced cells relative to the sictl-transfected cells. The viral protein level in the whole-cell lysates was analyzed by Western blotting with specific antibodies as indicated, and the expression of viral protein was remarkably enhanced in the GPS2-silenced cells (Fig. 2E and F). At the same time, we determined the effect of GPS2 silencing on cell viability in A549 cells and showed that there was no significant difference in cell activity of siGPS2-treated cells and mock-treated cells at 12, 24, and 36 h posttransfection (Fig. 2C and D).

We analyzed multiple-cycle growth kinetics in GPS2 knockout cells (A549^GPS2−/−^) by measuring progeny virus production to further define the role of GPS2 in the viral life cycle. Results showed that the progeny virus production at 24 and 36 h postinfection (hpi) in GPS2 knockout cells was approximately 1 log higher than that in the empty retrovirus-transduced control A549 cells (A549^KOCK^) (Fig. 2G). The effect of GPS2 on IAV replication was also monitored in A549 cells with HA-GPS2 stable expression (A549^GPS2+^). In the presence of high-level GPS2, the virus titers of WSN H1N1 viruses significantly decreased compared with empty retrovirus-transduced control A549 cells (Fig. 2H). Collectively, the GPS2 overexpression and knockout results indicated that GPS2 was a restriction factor in IAV replication.

### IAV infection induced degradation of GPS2 protein.

**FIG 3:**
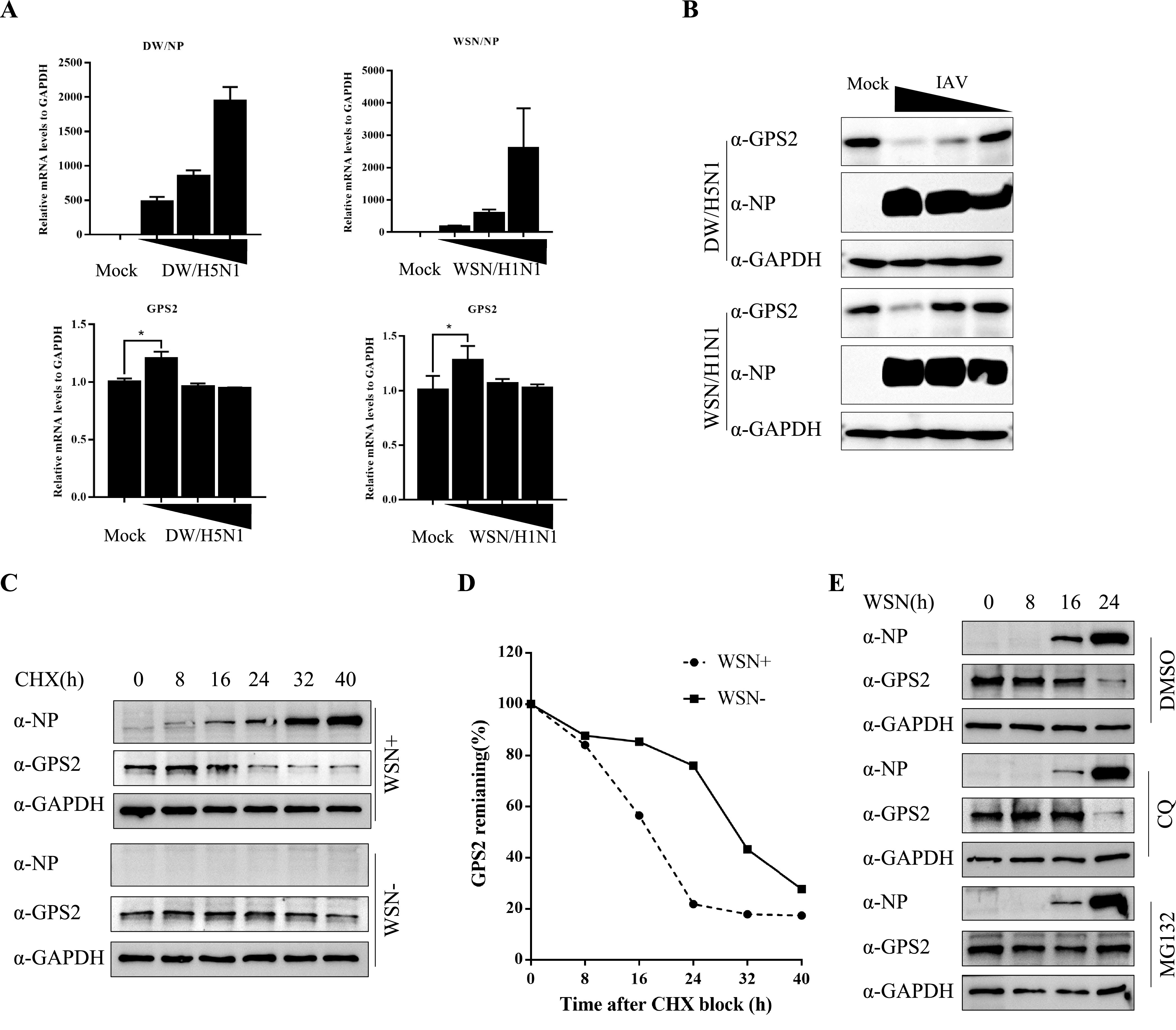
Influenza virus infection induced the degradation of GPS2 protein. (A and B) A549 cells were infected with A/WSN/33 (WSN/H1N1) or A/chicken/Hubei/327/2004 (DW/H5N1) at MOIs of 0, 0.01, 0.1, and 1, respectively. Samples were collected at 24 h postinfection. The levels of NP and GPS2 mRNA were determined by qRT-PCR. The viral RNA levels were normalized to the GAPDH level (means ± SD from three independent experiments) (*, *P* < 0.05; **, *P* < 0.01; all by two-tailed Student's *t* test) (A), and whole-cell lysates were assayed by Western blotting with the indicated antibodies. GAPDH was used as a loading control (B). (C) Half-lives of GPS2 in uninfected and infected A549 cells were examined. A549 cells uninfected or infected with WSN viruses (MOI = 1) were treated with CHX (100 μg/ml) at 6 h postinfection. At the indicated times after treatment, cells were harvested, and cell extracts were prepared for Western blotting to analyze GPS2 protein levels. (D) GPS2 levels shown in panel C were quantitated by band intensities analyzed using ImageJ (NIH) and normalized to GAPDH levels. (E) A549 cells infected with WSN viruses (MOI = 1) were treated with DMSO, MG132 (10 μM), or CQ (50 μM). At the indicated times, cells were harvested, and cell extracts were prepared for Western blotting using the indicated antibodies.

In previous studies, GPS2 could be degraded in the cells under human papillomavirus infection or other stimuli (33, 34). Thus, whether IAV infection reduced the protein level of GPS2 needed to be determined. A549 cells were infected with either the WSN H1N1 or the DW H5N1 virus, and then the mRNA and protein level of GPS2, as well as viral nucleoprotein, were measured. Results showed no obvious change in the GPS2 mRNA level in WSN H1N1- and DW H5N1-infected A549 cells (Fig. 3A), indicating that IAV infection did not alter the expression of GPS2 at the transcriptional stage. Analysis of the changes in GPS2 protein level after virus infection showed that the protein level of GPS2 decreased in the WSN H1N1- and DW H5N1-infected A549 cells in a dose-dependent manner (Fig. 3B).

The decrease in GPS2 protein level was not correlated with its mRNA level, so we next estimated the relative half-life of GPS2 protein in A549 cells infected or not infected with the WSN H1N1 virus. Cells were treated with cycloheximide (CHX) at 6 h postinfection and lysed after incubation at indicated time points, and the rates of GPS2 remaining in the cells were given as percentages of GPS2 protein level at the zero time point. As shown in Fig. 3C and D, the half-life of GPS2 was estimated to be approximately 32 h in uninfected control cells. Infection with the virus shortened the half-life of GPS2 to ∼16 h, suggesting that IAV infection promoted the protein degradation of GPS2 in the cells.

Two main pathways govern intracellular protein degradation: one is the proteasome pathway, and the other is the lysosome pathway (35). To determine how IAV infection causes the protein degradation of GPS2, the proteasome inhibitor MG132 and the lysosome inhibitor chloroquine (CQ) were used. We found that treatment with CQ or dimethyl sulfoxide (DMSO) did not reverse the effect of IAV infection on the GPS2 protein level in A549 cells. In contrast, treatment with MG132 almost completely prevented the IAV-induced protein degradation of GPS2 (Fig. 3E). These data suggested that IAV induced GPS2 degradation via the proteasome pathway and not via the lysosome pathway.

**FIG 4:**
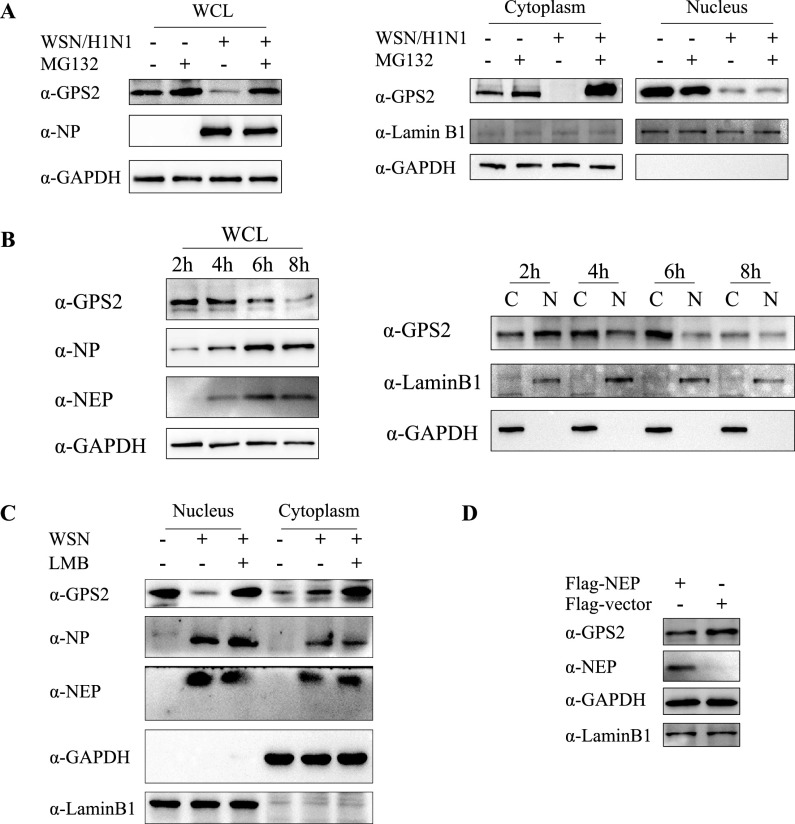
GPS2 degradation caused by IAV infection associated with NEP-mediated nuclear export. (A) A549 cells infected with A/WSN/33 (WSN/H1N1) at an MOI of 1 in the presence of MG132 (10 nM) and treated with DMSO as control. Cells were harvested at 24 hpi and subjected to nucleus and cytoplasmic fractions. (B) Wild-type A549 cells were infected with the A/WSN/33 (WSN/H1N1) at an MOI of 5. Samples were harvested at indicated time points postinfection and subjected to nucleus (N) and cytoplasmic (C) fractions. (C) A549 cells infected with A/WSN/33 (WSN/H1N1) at MOI of 1 in the presence of LMB (11 nM). Cells were harvested at 24 hpi and subjected to nucleus and cytoplasmic fractions. (D) A549 cells transfected with Flag-NEP or p3×Flag vector and harvested at 24 h posttransfection. Protein levels were assayed by Western blotting with the indicated antibodies. Lamin B1 was served as a nucleus loading control and marker, and GAPDH served as a cytosolic loading control and marker.

Considering that NEP and GPS2 interacted and that NEP was responsible for nuclear export during the IAV life cycle, we conducted an in-depth study on the degradation mechanism of GPS2. First, cytoplasmic and nucleus proteins were isolated from A549 cells infected with WSN H1N1 at an MOI of 1. Western blotting results showed that virus infection caused the degradation of GPS2, and the protein level of GPS2 in the cytoplasm bulk increased when virus-infected cells were treated with MG132 (Fig. 4A). Additionally, we also examined the protein level of GPS2 in the nucleus and cytoplasm of WSN H1N1-infected A549 cells at different time points in a replication cycle (2, 4, 6, and 8 hpi). Notably, the expression of NEP and the alteration of GPS2 subcellular localization occurred at 4 h postinfection, and GPS2 degradation happened at 8 h postinfection (Fig. 4B).

In the present study, we supposed that the NEP-GPS2 interaction mediated the nuclear export of GPS2 and stimulated its degradation. We initially found that the protein level of GPS2 in the nucleus was reversed when virus-infected cells were treated with leptomycin B (LMB); conversely, in the nuclei of WSN H1N1-infected cells without LMB treatment, the GPS2 protein level decreased (Fig. 4C). This finding suggested that the degradation of GPS2 was associated with chromosomal maintenance 1 (CRM1)-dependent nuclear export. We then examined the role of NEP, which was responsible for the nuclear export of vRNP and others during virus replication in this process. Results demonstrated that overexpressed NEP reduced the protein level of GPS2 in cells (Fig. 4D). Collectively, these data clearly demonstrated that the degradation of GPS2 protein caused by viral infection was related to NEP-mediated nuclear export.

### NEP mediated the nuclear export of GPS2 in IAV-infected cells.

**FIG 5:**
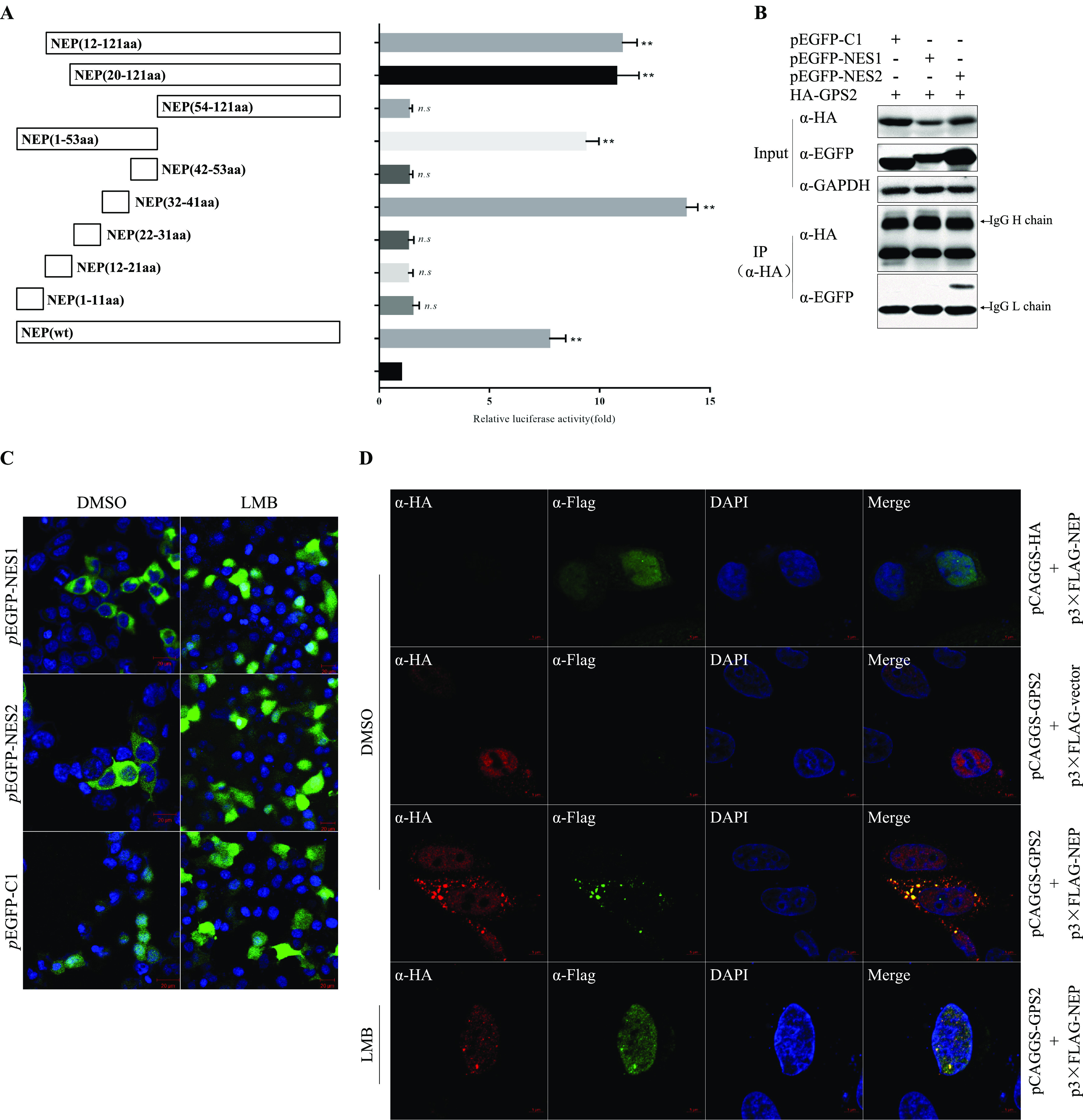
NEP mediated the nuclear export of GPS2. (A) Diagram of NEP truncated mutants, and the interaction strength with full-length GPS2 was assayed via MTH, respectively. Results are presented as the mean ± SD of three independent experiments (*, *P* < 0.05; **, *P* < 0.01; n.s., not significant; all by two-tailed Student's *t* test). (B) Interaction regions were confirmed by immunoprecipitation. NEP (aa 12 to 21) and NEP (aa 31 to 41) were chimeric expressed with EGFP. HEK293T cells were cotransfected with indicated plasmids, and cell lysates were immunoprecipitated and immunoblotted with specific antibodies as indicated. (C) NEP NES1 (aa 12 to 21) and NES2 (aa 31 to 41) were chimeric expressed with EGFP in pEGFP-C1 in the presence of DMSO or LMB in HeLa cells. Cells were fixed at 24 h posttransfection, and DAPI was used to stain the nucleus (blue). (D) Colocalization of GPS2 and NEP in transfected cells. HeLa cells cultured on slides were cotransfected with HA-GPS2 and/or Flag-NEP and treated with DMSO or LMB (11 nM) as indicated. HeLa cells were fixed at 24 h posttransfection and stained for HA-GPS2 (red) and NEP (green) by using the anti-HA mouse antibodies and anti-NEP rabbit antibodies, followed by immunostaining with Alexa Fluor 594-conjugated AffiniPure goat anti-mouse secondary antibodies and Alexa Fluor 488-conjugated AffiniPure goat anti-rabbit antibodies. DAPI was used to stain the nucleus (blue).

Based on previous results, we inferred that NEP may mediate the nuclear export of GPS2, but direct evidence is lacking. We initially confirmed that the interaction domain in IAV NEP was essential for the interaction between NEP and GPS2. NEP can be divided into a protease-sensitive N-terminal domain (amino acids [aa] 1 to 53) and a protease-resistant C-terminal domain (aa 54 to 121) (36). The N-terminal domain contained a nuclear export signal (NES) (aa 12 to 21) that mediated the interaction with the nuclear export protein CRM1 (13). The C-terminal structure was chiefly responsible for IAV M1 binding. On the basis of the domain composition of NEP, nine truncated mutants of NEP were constructed. The MTH assay was performed, and results revealed that NEP (aa 32 to 41) was the crucial domain responsible for the interaction with GPS2 (Fig. 5A). Moreover, we confirmed the MTH assay results by co-IP assay. As shown in Fig. 5B, NEP (aa 31 to 41) was indeed responsible for the interaction with GPS2 but not NEP (aa 12 to 21).

Previous studies have demonstrated that aa 12 to 21 in the N terminus of NEP (denoted as NES1) was a CRM1-dependent nuclear-export signal peptide and mediated the nuclear export of vRNPs (36). In the present study, aa 31 to 41 in the N terminus of NEP (denoted as NES2) was confirmed to be responsible for the interaction with GPS2, which mediated the nuclear export of the cargo in a CRM1-dependent manner (Fig. 5C). Thus, the subcellular localization of host protein GPS2 in the presence of NEP or virus infection required investigation. Flag-NEP and HA-GPS2 were transiently expressed and detected in HeLa cells to examine the subcellular localization of NEP and GPS2. Consistent with previous studies, transiently expressed GPS2 was primarily located in the nucleus of HeLa cells (29, 37) and then translocated into the cytoplasm and colocated in the cytoplasm upon the expression of NEP. However, GPS2 nuclear export was completely abolished when the cells were treated with LMB (Fig. 5D).

**FIG 6:**
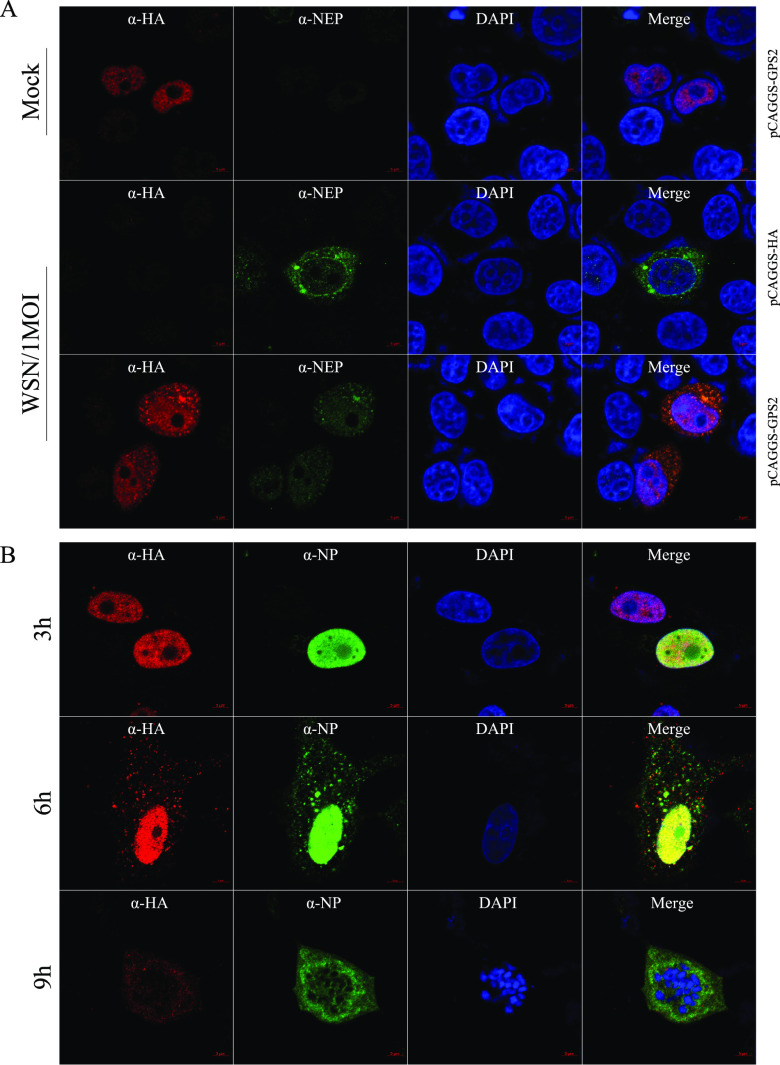
IAV infection mediated the nuclear export of GPS2. (A) HeLa cells cultured on slides were transfected with HA-GPS2 or pCAGGS-HA vector and then infected with A/WSN/33 (WSN/H1N1) at an MOI of 1 as indicated 12 h posttransfection. HeLa cells were fixed at 12 h postinfection and stained for HA-GPS2 (red) and NEP (green) by using anti-HA mouse antibodies and anti-NEP rabbit antibodies. (B) A549 cells cultured on slides were transfected with HA-GPS2 and then infected with A/WSN/33 (WSN/H1N1) at an MOI of 1 as indicated. At 3, 6, or 9 h postinfection, cells were fixed and stained for HA-GPS2 (red) and NP (green) by using anti-HA mouse antibodies and anti-NP rabbit antibodies, followed by immunostaining with Alexa Fluor 594-conjugated AffiniPure goat anti-mouse secondary antibodies and Alexa Fluor 488-conjugated AffiniPure goat anti-rabbit antibodies. DAPI was used to stain the nucleus (blue).

IAV infection can also mediate the nuclear output of GPS2. In HeLa cells, transiently expressed GPS2 was primarily located in the nucleus. Upon WSN H1N1 infection (12 hpi) with an MOI of 1, GPS2 nuclear export occurred, and it colocated with NEP in the cytoplasm (Fig. 6A). In GPS2-overexpressed A549 cells, we also studied the localization relationship between GPS2 and polymerase subunits. GPS2 colocalized with NP in the nucleus at 3 hpi when cells were infected with WSN H1N1 at an MOI of 1, and GPS2 was partially shifted out of the nucleus at 6 hpi. At 9 hpi, most of the GPS2 protein in the cell was located in the cytoplasm (Fig. 6B). These results collectively indicated that IAV infection can mediate the nuclear export of GPS2.

### GPS2 reduced IAV polymerase activity by binding to vRNPs.

**FIG 7:**
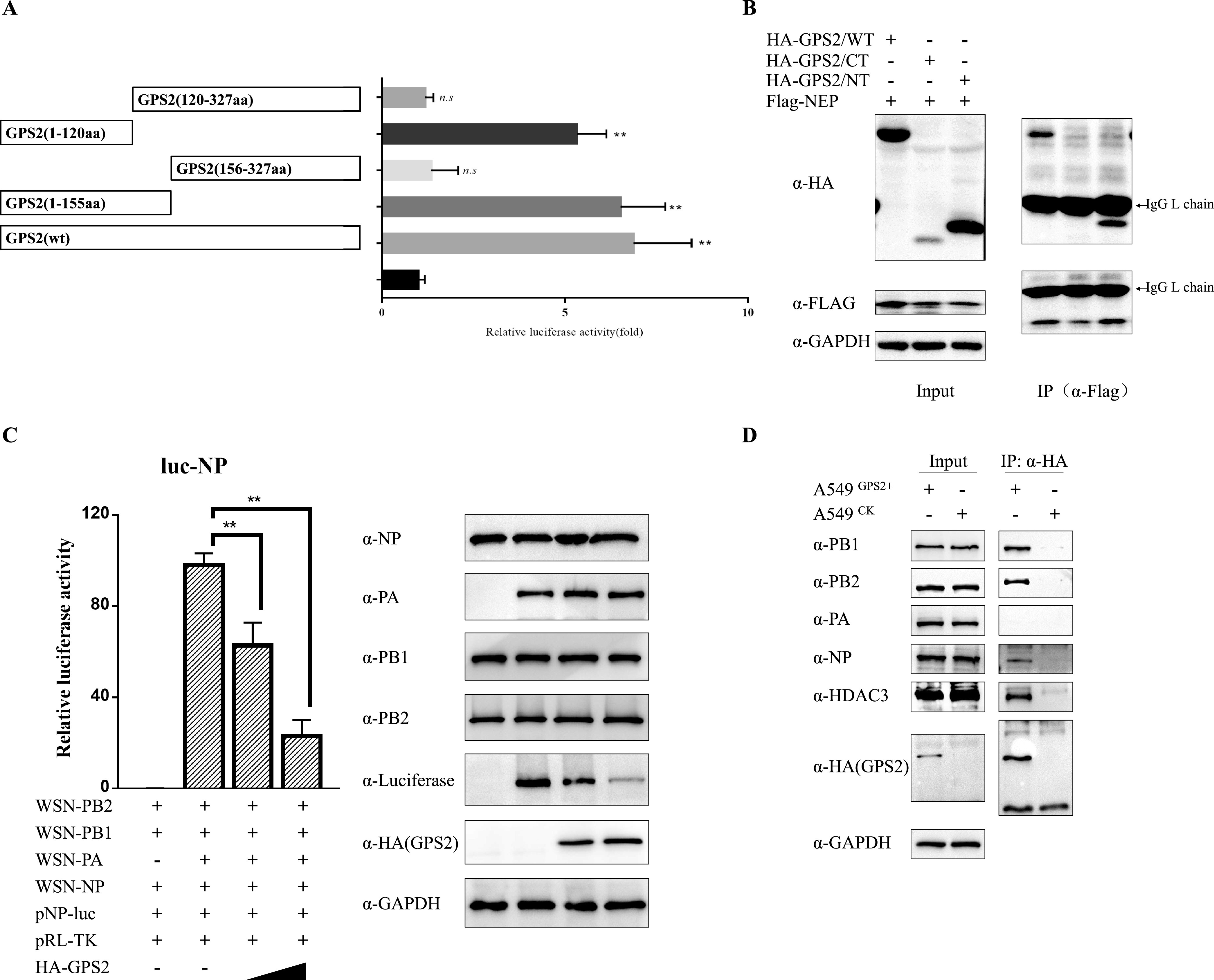
GPS2 inhibited IAV polymerase activity. (A) Diagram of GPS2 truncated mutants, and the interaction strength with full-length NEP was assayed via MTH, respectively. The results are presented as the mean ± SD of three independent experiments (*, *P* < 0.05; **, *P* < 0.01; n.s., not significant; all by two-tailed Student's *t* test). (B) Interaction regions were confirmed by immunoprecipitation. GPS2 was divided into N terminus (aa 1 to 155) and C terminus (aa 156 to 327). HEK293T cells were cotransfected with indicated plasmids, and the cell lysates were immunoprecipitated and immunoblotted with specific antibodies as indicated. (C) HEK293T cells were transfected with NP-luc and plasmids for the expression of viral PB1, PB2, PA, and NP proteins (A/WSN/1933 [H1N1]). Renilla luciferase was used as an internal control. Cells were also cotransfected with HA-GPS2 (0.2 and 1.0 μg). At 24 h posttransfection, luciferase activity was measured. Data represent the mean ± SD of three independent experiments. (**, *P* < 0.01 by two-tailed Student's *t* test). (D) Interactions between GPS2 and RNP components in virus-infected cells. A549^GPS2+^, or A549^CK^ cells infected with A/WSN/33 (WSN/H1N1) virus (MOI of 1) were lysed at 12 h postinfection. Co-IP analysis was performed using an anti-HA antibody, followed by Western blotting as indicated. Inherent interaction in the host cells between GPS2 and HDAC3 served as a positive control.

GPS2 consisting of 327 amino acids with a coiled-coil region in the N terminus (aa 1 to 120) mediates multi-interactions with other proteins (25, 29, 38). Moreover, the C terminus has many posttranslational modification sites (33, 39). We initially determined that the N-terminal coiled-coil domain of GPS2 was essential for NEP-GPS2 interaction (Fig. 7A and B). Considering that the coiled-coil region plays an important role in many transcription processes and GPS2 was a restriction factor in IAV replication, we further investigated its impact on influenza virus polymerase. For this purpose, a well-established mini-replicon assay system of WSN H1N1 was applied to examine the effect of GPS2 on the polymerase activity of WSN H1N1 virus. HEK293T cells were cotransfected with HA-GPS2, together with plasmids encoding PB1, PB2, polymerase acidic protein (PA), and NP, as well as a PolI-driven RNA expression plasmid encoding the NP vRNA segment (NP-luc). Results showed that GPS2 overexpression decreased polymerase activity in a dose-dependent manner, indicating that GPS2 negatively modulated IAV polymerase activity. The expression levels of the RNP components were measured to determine whether the GPS2-inhibited polymerase activity was due to a decrease in the expression levels of RNP subunits. Western blotting results showed that the expression levels of all RNP subunits, including PA, PB1, PB2, and NP, were unchanged (Fig. 7C), although the luciferase expression driven by PolI decreased in the GPS2-overexpressed cells. Additionally, co-IP assay results demonstrated that GPS2 could interact with proteins from RNPs (PB1, PB2, and NP) in IAV-infected cells (Fig. 7D). These results manifested that GPS2 negatively regulated IAV polymerase activity in a dose-dependent manner, and it did not reduce the expression of RNP subunits in the mini-replicon assay.

### GPS2 participated in IAV genome replication by altering RNA synthesis.

**FIG 8:**
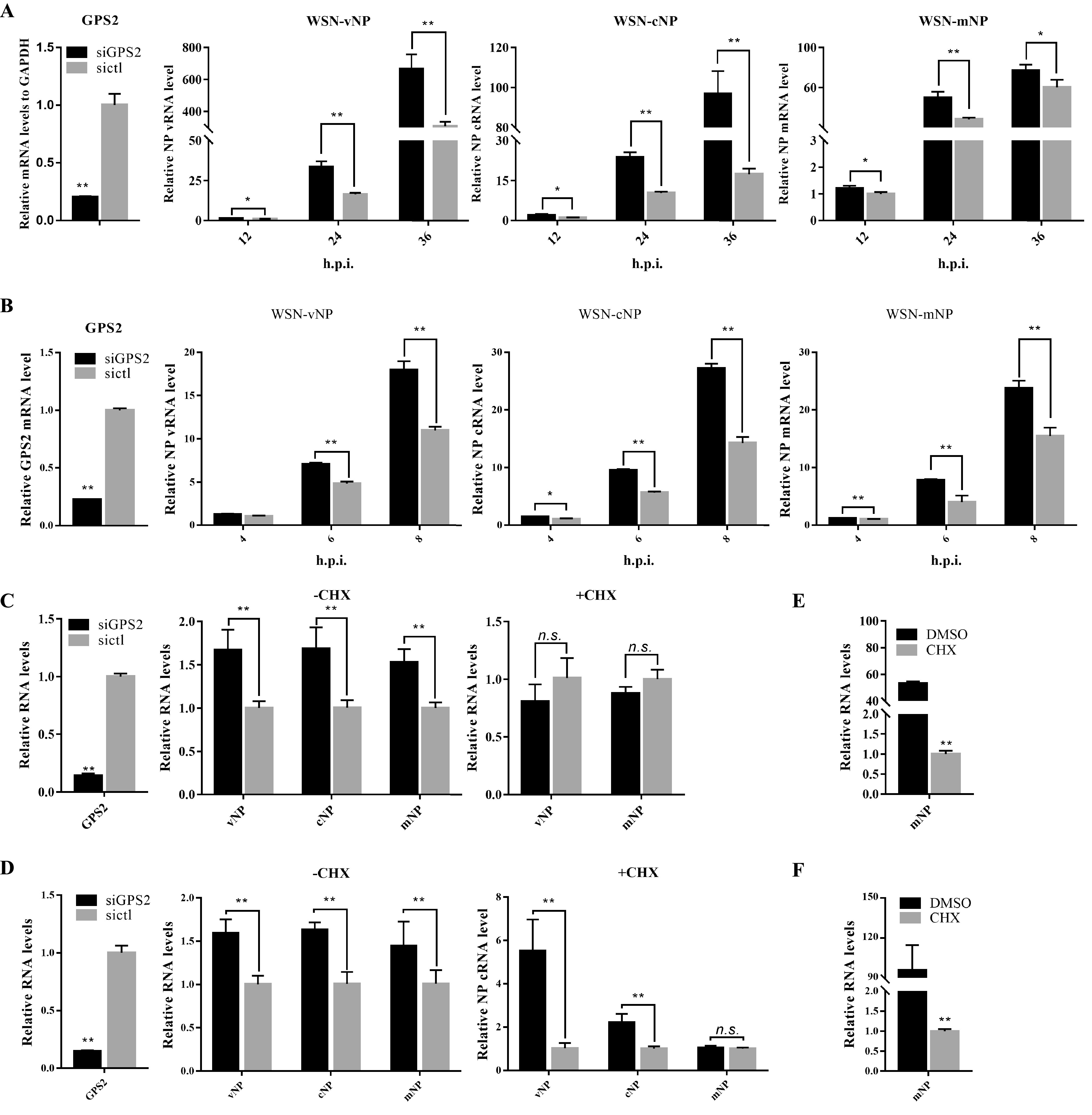
Effect of GPS2 on IAV RNA synthesis. A549 cells were transfected with siGPS2 or negative-control sictl and then infected with A/WSN/33 (WSN/H1N1) virus (MOI of 0.01). (A and B) Samples were collected at the indicated times. Levels of NP RNAs (vRNA, cRNA, and mRNA) were determined by qRT-PCR. (C and D) Cells were pretreated with 100 μg/ml CHX 1 h before infection and then infected with A/WSN/33 (WSN/H1N1) virus (MOI of 0.01) (C), or cells treated with 100 μg/ml CHX 2 h after incubation with A/WSN/33 (WSN/H1N1) virus (MOI of 0.01) (D). Samples were collected at 8 hpi. Levels of NP RNAs (vRNA, cRNA, and mRNA) were determined by qRT-PCR. Viral RNA levels were normalized to the GAPDH level. (E and F) Comparison of NP mRNA levels in cells infected with virus for 8 h in the presence or absence of CHX (mean ± SD from three independent experiments) (*, *P* < 0.05; **, *P* < 0.01; all by two-tailed Student's *t* test).

GPS2 inhibited IAV polymerase activity, suggesting that GPS2 may affect influenza virus RNA synthesis during influenza virus replication. GPS2-silenced A549 cells were infected with the WSN H1N1 virus, and the levels of vRNA, cRNA, and mRNA specific for the NP segment were examined by real-time PCR with specific primers. Reverse transcriptase quantitative PCR (RT-qPCR) analysis results showed that the viral RNA levels of all three species increased in the GPS2-silenced A549 cells compared with control cells at 12, 24, and 36 hpi (Fig. 8A). This finding was consistent with the negative regulation of IAV polymerase activity by GPS2 in a mini-replicon assay system.

Considering that IAV uses different strategies for transcription and RNA replication, we next investigated the effects of GPS2 knockdown on viral RNA transcription and replication. A previous study has shown that the replication and not the transcription of IAV RNA requires newly synthesized viral proteins (40). Cycloheximide (CHX), an inhibitor of protein synthesis in eukaryotic cells, was used to block the synthesis of viral proteins and host proteins. As a result, the synthesis of cRNA and the replication of viral RNA were inhibited, but the levels of primary transcription from incoming vRNPs were unaffected (41, 42).

In GPS2-silenced A549 cells without CHX (−CHX), the vRNA, cRNA, and mRNA levels significantly increased at 6 and 8 hpi compared with those in the control cells (Fig. 8B). In the presence of CHX, no significant difference in vRNA and mRNA levels was found between GPS2-silenced and control A549 cells at 8 hpi, and cRNA could not be detected (Fig. 8C). These data indicated that GPS2 exerted no effect on the synthesis of viral mRNA; however, the data did not directly demonstrate that GPS2 affected the replication of the viral genome. A direct reason may be that the newly synthesized cRNA was degraded by nuclear nuclease because no newly synthesized polymerase subunits were available to form a stable RNP.

The experimental results changed when the treatment of CHX was adjusted 2 h after WSN H1N1 was incubated on the cell and the other experimental settings remained unchanged. cRNA could be detected, although its amount was significantly lower than that in the experimental group without CHX. Between GPS2-silenced and mock-silenced A549 cells, WSN-NP mRNA levels showed no obvious difference, whereas the WSN-NP vRNA and cRNA levels significantly increased (Fig. 8D). These results suggested that GPS2 was involved in IAV genome replication. The NP mRNA levels in the CHX-treated and untreated infected cells were determined to verify the inhibition of CHX treatment on virus multiplication. Results showed that NP mRNA levels were reduced approximately 60-fold after CHX treatment (Fig. 8E and F). Taken together, these findings demonstrated that GPS2 reduced RNA synthesis in RNA replication but exerted no effect on primary transcription.

### GPS2 reduced IAV polymerase activity by interfering with vRNP assembly.

**FIG 9:**
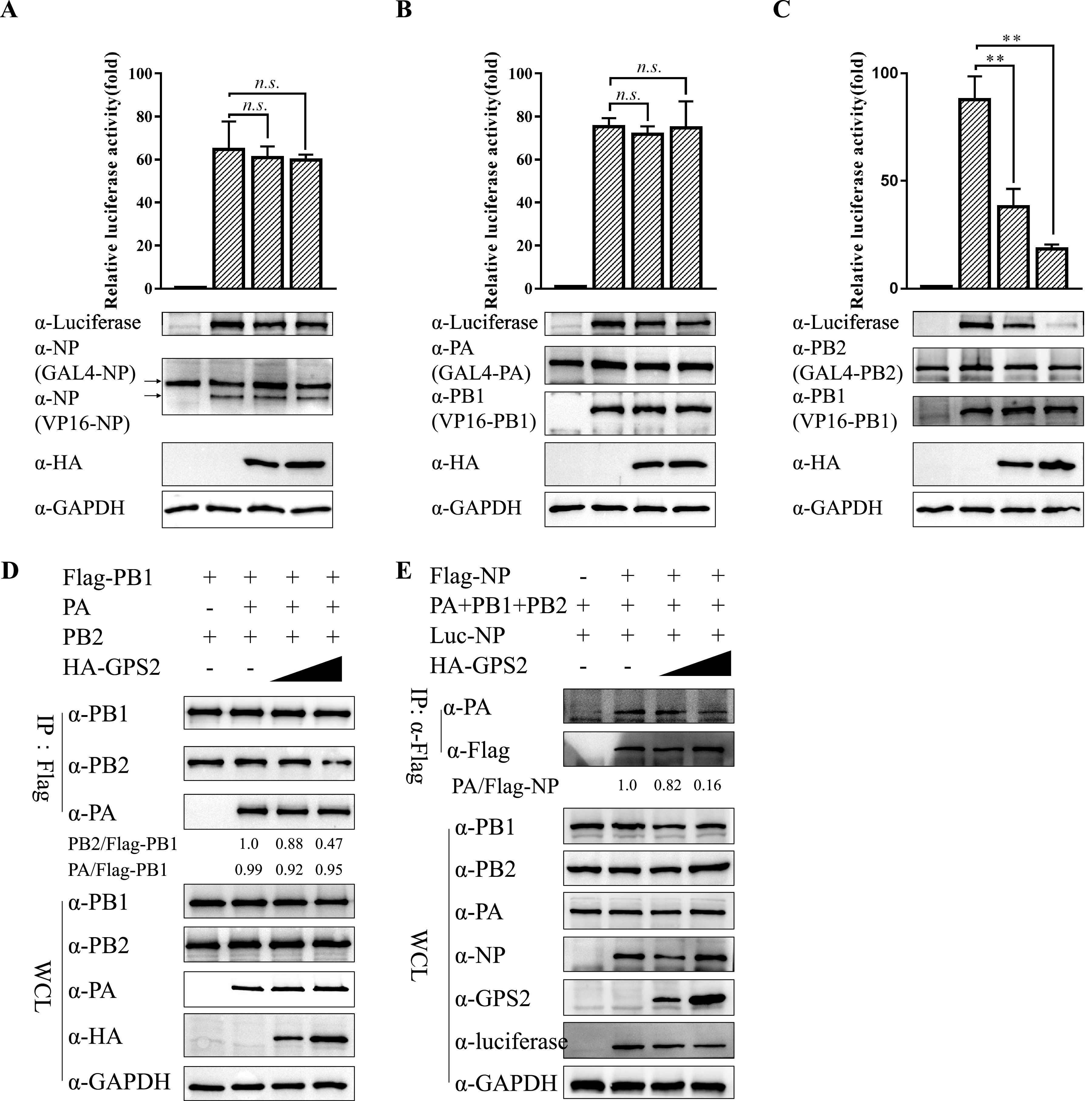
GPS2 interfered with IAV vRNP assembly. (A to C) Effect of GPS2 on NP-NP, PA-PB1, or PB2-PB1 interaction in MTH assay. HEK293T cells transfected with pG5luc and plasmids for expression of GAL4 with NP, PA, or PB2, and VP16 with NP or PB1. Renilla luciferase served as an internal control. Cells were also cotransfected with HA-GPS2 (0.5 and 1.0 μg). At 24 h posttransfection, luciferase activity was measured. The expression levels of individual proteins in the lysed supernatant were analyzed by Western blotting. (D) Effect of GPS2 on 3P formation. HEK293T cells were transfected with pCDNA3.1-PA, pCDNA3.1-PB2, HA-GPS2 (0.2 and 1.0 μg), and Flag-PB1. Cells were then treated as described above, and co-IP was performed using an anti-Flag antibody. Western blotting was performed using antibodies as indicated. Band intensities were quantified, and relative precipitated PA/Flag-PB1 and PB2/Flag-PB1 ratios are shown below. (E) Effect of GPS2 on vRNP assembly. HEK293T cells were transfected with vRNP reconstitution plasmids together with HA-GPS2 (0.2 and 1.0 μg), and then co-IP was performed using an anti-Flag antibody followed by Western blotting. Relative precipitated PA/Flag-NP ratios are shown below. For all experiments, data represent the mean ± SD of three independent experiments. (**, *P* < 0.01 by two-tailed Student's *t* test). Band intensities were analyzed using ImageJ (NIH). GAPDH served as a loading control.

IAV genome replication depends on the assembly of progeny vRNPs by newly synthesized polymerase subunits and oligomerized NP in the nucleus (43). Accordingly, we evaluated whether GPS2 participated in viral RNP assembly, including 3P formation and viral RNP (viral RNA, NP, and 3P) assembly. To our knowledge, the vRNP assembly is the process of forming biologically active heteropoly through pair interaction between polymerase subunits in the nucleus, such as NP-NP, PA-PB1, and PB2-PB1 (44–46). Therefore, whether GPS2 interfered with the pair interaction between polymerase subunits was necessary to investigate, and the MTH assay was applied to quantify it. Results showed that GPS2 could reduce the interaction between PB1 and PB2, but not that between NP-NP and PA-PB1, in a dose-dependent manner (Fig. 9A to C).

Next, the effect of GPS2 on 3P formation was examined by co-IP, and Flag-PB1 was used as bait to immunoprecipitate PA and PB2. We showed that PA was precipitated by PB1 equally with or without HA-GPS2, whereas PB2 decreased in the presence of GPS2 (Fig. 9D), demonstrating that GPS2 affected 3P formation. NP directly interacted with PB1 and PB2 but not with PA, and the NP-PA interaction occurred only in the context of a vRNP (44). Therefore, we used a vRNP reconstitution system in which the NP was Flag tagged to form the functional viral RNPs and subsequently performed a co-IP experiment by using an anti-Flag antibody. The amount of PA coprecipitated by Flag-NP represents the efficiency of vRNP assembly. Data showed that the amount of PA precipitated by Flag-NP significantly decreased in a dose-dependent manner. The expression of luciferase driven by vRNP also decreased in GPS2-overexpressed cells, whereas the expression of all RNP components was almost unchanged (Fig. 9E). This result suggested that GPS2 directly reduced vRNP assembly. Taken together, these findings provided evidence that GPS2 reduced IAV polymerase activity by interfering with vRNP assembly.

### NEP weakened the inhibition of GPS2 to viral polymerase activity.

**FIG 10:**
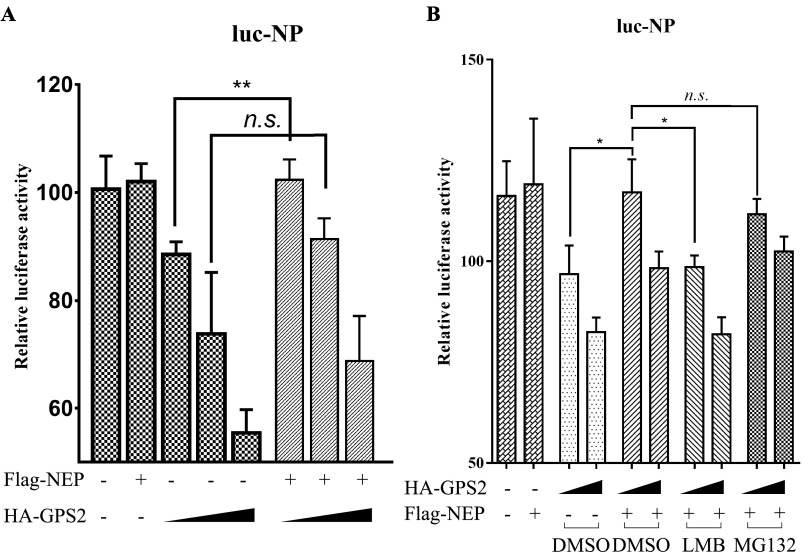
NEP weakened the inhibition of GPS2 to viral polymerase activity. (A) HEK293T cells were cotransfected with NEP (100 ng) and GPS2 (100, 200, or 500 ng), together with plasmids encoding PB1, PB2, PA, and NP, as well as a PolI-driven RNA expression plasmid encoding the NP vRNA segment (NP-luc) as indicated. At 24 h posttransfection, luciferase activity was measured. (B) HEK293T cells were cotransfected with HA-GPS2 (100 or 200 ng) and Flag-NEP (100 ng), together with plasmids encoding PB1, PB2, PA, and NP, as well as a PolI-driven RNA expression plasmid encoding the NP vRNA segment (NP-luc), as indicated. Cells were treated with LMB, MG132, or DMSO as indicated 8 h posttransfection, and luciferase activity was measured 24 h later. For all experiments, data represent the mean ± SD of three independent experiments. (*, *P* < 0.05; **, *P* < 0.01; n.s., not significant; all by two-way analysis of variance [ANOVA] test).

To further extend our observations, we tested the effects of NEP on the GPS2-inhibited polymerase activity of IAV. NEP alone can reportedly shift the polymerase activity through a complex process (18). First, the transfection dose of NEP (100 ng) was determined without affecting the activity of polymerase. NEP (100 ng) and GPS2 (100, 200, or 500 ng) were then cotransfected in HEK293T cells. At 24 h posttransfection, luciferase activity was measured. As shown in Fig. 10A, GPS2 inhibited IAV polymerase activity in a dose-dependent manner without NEP. When cotransfected with 100 ng of NEP and 100 ng of GPS2, the inhibition effect to IAV polymerase activity caused by GPS2 was reduced. When HEK293T cells were transfected with more GPS2 (200 or 500 ng), the expression of NEP (100 ng) or not did not significantly change the inhibition effect to IAV polymerase activity caused by GPS2. This finding may have been due to an overabundance of GPS2. Collectively, these results provided evidence that the inhibition of GPS2 on IAV polymerase activity was independent of NEP.

Moreover, the effects of LMB and MG132 on the inhibition of GPS2 to viral polymerase activity were studied because LMB and MG132 could block the degradation of GPS2 in IAV-infected cells. HEK293T cells were cotransfected with HA-GPS2 (100 or 200 ng) and Flag-NEP (100 ng), together with plasmids encoding PB1, PB2, PA, and NP, as well as a PolI-driven RNA expression plasmid encoding the NP vRNA segment (NP-luc). Cells were treated with LMB, MG132, or DMSO as indicated 8 h posttransfection, and then luciferase activity was measured 24 h later. MG132 treatment did not interfere with the rescue of luciferase activity by NEP between groups transfected with the same dose of GPS2, but LMB blocked it (Fig. 10B). These findings demonstrated that NEP weakened the inhibition of GPS2 to viral polymerase activity by interacting with GPS2 and mediating the nuclear export of GPS2.

## DISCUSSION

NEP is coded by the nonstructural (NS) segment of IAV through RNA splice, also known as NS2. During the life cycle of IAV, new synthetic viral proteins such as PB2, NP, and PB1-PA heterodimers are transported into the nucleus to form new viral RdRp (45). In the nucleus, viral RNA transcription and replication occur in a viral RdRp-dependent manner (47). Newly synthesized vRNPs, together with M1, NEP CRM1, and other factors, are then trafficked out of the nucleus (32, 48). Our study demonstrated that GPS2 could reduce the interaction between PB1 and PB2 and interfere with vRNP assembly. Therefore, GPS2 inhibited the viral RNA synthesis and negatively regulated the replication of IAV. Furthermore, IAV NEP interacted with GPS2 and mediated the nuclear export of GPS2, thereby activating the degradation of GPS2 protein. Thus, NEP-GPS2 interaction weakened the inhibition of GPS2 to viral polymerase activity, benefiting virus replication.

GPS2 contains an N-terminal coiled-coil domain (aa 12 to 101), which is essential for interaction with other proteins. GPS2 interacts with SMRT/nuclear receptor corepressor (N-CoR) and transducin β-like 1 (TBL1) through its coiled-coil domain in the N terminus, and SMRT and GPS2 are both protected from proteolysis when in complex with TBL1 (33, 38). In the present study, we demonstrated that human GPS2 interacted with IAV NEP through its N-terminal coiled-coil domain.

Considering that the coiled-coil domain in the N terminus mediates many host cell-intrinsic interactions associated with GPS2, closely related to its biological function and protein stability, we first studied the protein levels change in IAV-infected cells. Interestingly, WSN H1N1 or DW H5N1 virus infection could lead to protein level reduction, and further research showed that the mechanism is the interaction between NEP and GPS2, which mediated the nuclear export of GPS2 and promoted its degradation.

Previous studies have demonstrated that a fine balance between protein stabilization and degradation tightly regulates GPS2 nuclear function. On one hand, the methylation of GPS2 by arginine methyltransferase PRMT6 at Arg-312/323 strengthens the interaction with TBL1 and inhibits the proteasome-dependent degradation of GPS2. On the other hand, when the TBL1 protective role is removed or the methylation level of GPS2 decreases, the E3 ligase Siah2-mediated ubiquitination of GPS2 at K254/300/327 occurs and is accompanied by the degradation by 26S proteasome (33, 37, 49). Interestingly, as shown in Fig. 4C, the GPS2 protein level in both cytoplasm and nucleus significantly increased when we treated IAV-infected A549 cells with LMB. To our knowledge, another posttranslational modification of GPS2 associated with protein subcellular localization and stability may have occurred during this process and is thus worthy of further study (50).

In the nucleus, GPS2 acts as a core subunit of a fundamental chromatin-modifying corepressor complex containing histone deacetylase 3 (HDAC3) and NCoR/SMRT (23–25, 51, 52). For example, the interaction between E8^E2C proteins from human papillomavirus 1 (HPV1), HPV8, HPV16, and HPV31 and the NCoR/SMRT corepressor core complex consisting of GPS2 and other components mediates the transcriptional repression and inhibition of E1/E2-dependent replication, thereby revealing a novel, highly conserved role for the cellular NCoR/SMRT-corepressor complex in the control of HPV replication (25). Moreover, GPS2 is a protein partner for human papillomavirus E6 proteins, and GPS2-E6 interaction could cause GPS2 degradation *in vivo* (34). GPS2 is inactivated or sequestered by viral proteins as a potential means to promote virus replication by affecting the GPS2- and N-CoR-mediated pathways (23, 34).

Coincidentally, HPV and IAV are viruses that replicate in the nucleus. The synthesis of influenza virus vRNA, mRNA, and cRNA is inseparable from the involvement of host factors. No conclusive evidence is available regarding the involvement of host NCoR/SMRT-corepressor complex in influenza virus replication, but our data demonstrated that GPS2 negatively regulated polymerase assembly and activity in the nucleus. We have demonstrated that NEP weakened the inhibition of GPS2 to viral polymerase activity in the mini-replicon assay and suggested that GPS2 was also inactivated or sequestered by IAV NEP. Collectively, IAV NEP served as an adaptor and removed the inhibition of GPS2 to viral polymerase activity. Additionally, we attempted to investigate what was the main role of NEP in this process. Did it mediate the nuclear export of GPS2 or GPS2 degradation? Our data suggested that MG132 did not interfere with NEP-mediated rescue of luciferase activity, but LMB blocks it. As shown in Fig. 4C, LMB could block the nuclear export of GPS2 upon IAV infection, as well as its degradation. But MG132 could only inhibit protein degradation and not include GPS2 nuclear output (Fig. 4A). Therefore, we inferred that GPS2 could function as a negative regulator to IAV polymerase only in the nucleus.

To our knowledge, influenza virus polymerase is a heterotrimeric complex consisting of PA, PB1, and PB2, with multiple enzymatic and ligand-binding activities allowing the synthesis of capped, polyadenylated mRNAs during transcription and full-length genomic/antigenomic RNAs during replication (6). PB1 is located in the center of the influenza virus polymerase, which connects PB2 and PA to form a complete 3P complex. The N-terminal domain of PA displays the endonuclease activity needed for cap snatching, and PB1 hosts the polymerase catalytic active site, as well as specific binding sites, for the conserved 5′ and 3′ vRNA termini (53, 54). Different posttranslational modifications of 3P affect polymerase assembly and activity without changing the protein levels of 3P (55, 56). In the present study, we indicated that GPS2 inhibited PB1-PB2 interaction and the IAV polymerase assembly. Previous studies have shown that GPS2 is an epigenome modulator. On one hand, GPS2 regulating tumor necrosis factor alpha (TNF-α) signaling and lipid metabolism in adipose tissue through the modulation of K63 ubiquitination events based on the direct inhibition of Ubc13 enzymatic activity (29). Some studies have also suggested that GPS2 could mediate the inhibition to ubiquitin ligase RNF8 and the stabilization inhibition and stabilization of the H3K9 histone demethylase DM4A/JMJD2 (57). Affecting the posttranslational modification of polymerase subunits may be an intrinsic mechanism for GPS2 in inhibiting the assembly of influenza virus polymerase, but more studies are needed to support it.

Overall, our results confirmed that the direct interaction of GPS2 with NEP occurred through a newly recognized NES of NEP. In agreement with its proposed functions, GPS2 was observed inside and outside the nucleus. The control of GPS2 localization may be an important determinant of its molecular functions. Our findings revealed that IAV infection led to decreased GPS2 in the nucleus by interacting with NEP, blocking the inhibition to the viral polymerase activity and vRNP assembly. Altogether, these findings provided evidence that NEP-GPS2 interaction weakened the inhibition of GPS2 to viral polymerase activity, benefiting virus replication.

## MATERIALS AND METHODS

### Cells and viruses.

Human adenocarcinoma alveolar basal epithelial (A549) cells, human embryonic kidney 293T (HEK293T) cells, and Madin-Darby canine kidney (MDCK) cells were maintained in F12 (HyClone, Beijing, China), Dulbecco’s modified Eagle medium (DMEM), and RPMI-1640 (Gibco, New York, NY, USA) medium supplemented with 10% fetal bovine serum and then cultured at 37°C under 5% CO_2_.

IAV H1N1 (A/WSN/1933 [WSN H1N1]) virus was obtained by reverse genetics as described by Hoffmann and Webster (58) and maintained by our laboratory. WD-Flag-NEP virus was obtained by reverse genetics in the context of WSN H1N1 background, expressing HA, NA, and NS from DW H5N1 virus. Furthermore, the NS segment was modified by expressing an NS2 fused with a 2×Flag tag without affecting NS1 expression (32, 59). The H5N1 virus strain A/chicken/Hubei/327/2004 (H5N1) (abbreviated as DW) isolated from chicken and stored by State Key Laboratory of Agricultural Microbiology was cultured in specific-pathogen-free (SPF) chicken embryo and stored at −80°C (60). All viruses were amplified using 10-day-old embryonic chicken eggs and then titrated by determining log_10_ TCID_50_/ml values on MDCK cells. All experiments with the H5N1 virus were performed in a biosafety level 3 laboratory (BSL-3). This study was performed in accordance with the recommendations of the BSL-3 laboratory at Huazhong Agricultural University (HZAU). All procedures were approved by the Intuitional Biosafety Committee of HZAU.

### Plasmids.

To construct pCAGGS-HA-GPS2 (HA-GPS2), the full-length cDNA of GPS2 amplified by PCR was cloned into pCAGGS-HA vector digested by EcoRI/XhoI. To construct p3×Flag-NEP (Flag-NEP) (A/chicken/Hubei/327/2004 [H5N1]), the full-length cDNA of NEP amplified by PCR was cloned into p3×Flag vector (Flag) digested by EcoRI/KpnI. The Flag-tagged, HA-tagged, and nontagged pcDNA3.1 plasmids encoding PB1, PB2, PA, and NP were derived from the H1N1 (A/WSN/1933 [H1N1]) virus.

The NEP open reading frame (ORF) from IAV (A/chicken/Hubei/327/2004 [H5N1]) was cloned into pGBKT7 (pGBKT7-NEP) as bait for yeast two-hybrid (Y2H) screening and pGEX-6p-1 (pGEX-NEP) for a glutathione *S*-transferase (GST) pulldown assay. For mammalian two-hybrid assays, the full length of NEP and the truncated regions corresponding to NEP (aa 2 to 53), NEP (aa 54 to 121), NEP (aa 12 to 121), NEP (aa 20 to 121), NEP (aa 2 to 11), NEP (aa 22 to 31), NEP (aa 32 to 41), and NEP (aa 42 to 53) were cloned into the SalI*/*NotI sites of the pBind vector, whereas the full length of GPS2 was cloned into the XbaI/KpnI sites of the pACT vector. NES1 (aa 12 to 21) and NES2 (aa 31 to 41) were cloned into the C terminus of enhanced green fluorescent protein (EGFP) in pEGFP-C1 by overlapping PCR. All constructs were verified by sequencing.

### Antibodies.

The antibodies used for Western blotting, immunoprecipitation, and indirect immunofluorescence were anti-Flag mouse monoclonal antibody (catalog no. F3165; Sigma, USA); anti-GPS2 and anti-HA rabbit polyclonal antibodies (catalog nos. A3901 and AE036, respectively; Abclonal, China); anti-HA, anti-GFP, anti-glyceraldehyde-3-phosphate dehydrogenase (GAPDH), anti-TBL1, and anti-lamin B1 mouse monoclonal antibodies (catalog nos. 66006-2-Ig, 66002-1-Ig, 60004-1-Ig, 66955-1-Ig, and 66095-1-Ig, respectively; Proteintech, USA); rabbit polyclonal antibodies against IAV proteins PB1, PB2, PA, NP, and NEP (catalog nos. GTX125923, GTX125926, GTX118991, GTX125989, and GTX125928, respectively; GeneTex, USA); and Alexa Fluor 488-conjugated AffiniPure goat anti-rabbit and Alexa Fluor 594-conjugated AffiniPure goat anti-mouse secondary antibodies.

The small-molecule compounds used in this study were cycloheximide (CHX), 4′,6-diamidino-2-phenylindole (DAPI), chloroquine (CQ), and leptomycin B (LMB).

### Small interfering RNAs and transfection.

The sequence of siRNA oligonucleotides used in this study targeting human GPS2 was 5′-CCUGCAGUGCAGUACCUAUTT-3′ (29). siRNA oligonucleotides were chemically synthesized and transfected into 293T or A549 cells by using the Lipofectamine 2000 reagent in accordance with the manufacturer’s instructions. In a typical procedure, plasmids, siRNAs, and Lipofectamine were diluted to equal volumes with Opti-MEM and incubated for 5 min at room temperature. The diluted Lipofectamine and the diluted DNA (or RNA) were mixed and incubated for 20 min at room temperature. The mixture was added to the cells and incubated for 6 h, and then the cells were cultured in a fresh medium supplemented with 10% fetal bovine serum (FBS).

### RNA isolation and quantitative real-time PCR analysis.

For quantitative reverse transcription-PCR (qRT-PCR), cells were lysed with TRIzol reagent, and total RNA was extracted in accordance with the manufacturer’s instructions. A 2-µg aliquot of RNA was used for first-strand cDNA synthesis through reverse transcription. Real-time PCR was performed using FastStart Universal SYBR green master mix. The cycle conditions included 2 min at 50°C, 10 min at 95°C, and 40 cycles of amplification for 15 s at 95°C and 1 min at 60°C.

The levels of viral NP vRNA, cRNA, and mRNA were determined using a previously described strand-specific real-time RT-PCR method (61). The primer sequences used for the reverse transcription were cRNA, 5′-CCTTGTTTCTACT-3′; oligo(dT)18(mRNA), TTTTTTTTTTTTTTTTTT; and uni12(vRNA), AGCAAAAGCAGG. Subsequently, the following primers were used for qPCR: 5′-CAATTCCAAGGCAGTCCTG-3′ (forward) and 5′-TCTGAGTGGGCTGAAAGTG-3′ (reverse) for human GPS2 gene, 5′-GCTAAGGCTGTGGGCAAGG-3′ (forward) and 5′-GGAGGAGTGGGTGTCGCTG-3′ (reverse) for human GAPDH gene, 5′-AACGACCGGAATTTCTGGAGAGG-3′ (forward) and 5′- CCGTACACACAAGCAGGCAAGC-3′ (reverse) for DW H5N1 virus NP gene, and 5′- GCCTGCCTGCCTGTGTGTATGGAT-3′ (forward) and 5′- GGCATGCCATCCACACCAGTTGAC-3′ (reverse) for WSN H1N1 virus NP gene.

### GST pulldown and co-IP assays.

At 36 h after expression of HA-GPS2 in HEK293T cells, the cells were lysed using lysis buffer (50 mM Tris [pH 8.0], 100 mM NaCl, 20 mM NaF, 50 mM KH_2_PO_4_, 1% Triton X-100, 10% glycerol, and 0.1 mM dithiothreitol [DTT]) containing 1 mM phenylmethylsulfonyl fluoride (PMSF; Amresco, OH, USA). For co-IP, the lysate supernatant was diluted in binding buffer (50 mM Tris-HCl [pH 8.0], 100 mM KCl, 0.1 mM EDTA, 0.2% NP-40, 2.5% glycerol, and 1 mM DTT) and then incubated with 1 μg of an anti-Flag tag antibody and A/G Plus-agarose (Santa Cruz Biotechnology, USA) for 2 h at 4°C. The beads were washed and then resuspended in sodium dodecyl sulfate (SDS) loading buffer. The bound proteins were resolved by SDS-PAGE and Western blot analysis.

For GST pulldown, the GST-NEP and GST proteins were expressed in Escherichia coli BL21(DE3) and bound to glutathione-Sepharose 4B for 2 h at 4°C, respectively. The same procedure as co-IP was performed except that the cell lysate was incubated with beads for 6 h at 4°C.

### Immunofluorescence assay and confocal microscopy.

Indirect immunofluorescence and confocal microscopy were performed as previously described (48). In a typical procedure, HeLa or A549 cells were fixed with 4% paraformaldehyde for 10 min, treated with 0.2% (vol/vol) Triton X-100 for 10 min, and then incubated with 1% (wt/vol) bovine serum albumin for 1 h at room temperature. Samples were then incubated with the indicated primary antibody for 2 h, incubated with the appropriate Alexa Fluor-conjugated secondary antibody, and then stained with DAPI to visualize DNA. Images were acquired using a confocal microscope.

### Polymerase activity assay.

After reaching 70% confluence in a 12-well plate, HEK293T cells were transfected with the corresponding plasmids by using the transfection reagent Lipofectamine 2000 in a nonserum medium in accordance with the manufacturer’s instructions. Firefly luciferase activity was normalized to Renilla luciferase activity as the relative activity in accordance with the protocol.

### Nuclear and cytoplasmic fractionation.

Subcellular fractions were extracted as previously described (50). A total of 10^6^ A549 cells treated accordingly were harvested and lysed with 100 μl of cytoplasmic extraction buffer (10 mM HEPES pH 7.9, 1 mM EDTA, 210 mM mannitol, 70 mM sucrose, 10 mM *N*-ethylmaleimide [NEM], 50 mM NaF, 2 mM Na_2_VO_3_, 1 mM PMSF, and protease inhibitor cocktail). The cell suspension was incubated on ice for 5 to 10 min and vortexed vigorously for 20 to 30 s and then centrifuged at 16,000 × *g* for 30 s. The nuclear pellet was incubated for 20 min in high-salt buffer (10 mM HEPES, pH 7.9, 20% glycerol, 420 mM NaCl, 1.5 mM MgCl_2_, 0.2 mM EDTA, 0.5 mM DTT, 10 mM NEM, 50 mM NaF, 2 mM Na_2_VO_3_, 1 mM PMSF, and protease inhibitor mix) and then centrifuged at 16,000 × *g* for 5 min.

### Western blot analysis.

Cell lysates were prepared by washing the transfected or IAV-infected cells with precooled phosphate-buffered saline (10 mM Na_2_HPO_4_, 1.7 mM NaH_2_PO_4_, and 140 mM NaCl, pH 7.4) and extracting the total protein by using cell lysis buffer (50 mM Tris-HCl, 150 mM NaCl, 1% Triton X-100, 0.5% sodium deoxylcholate, 0.1% SDS, 10 mM NaF, 1 mM EDTA, pH 7.4, 1 mM PMSF, and protease inhibitor mix). The extracted proteins were separated by SDS-PAGE and transferred onto a nitrocellulose filter membrane. After blocking, the membrane was incubated with primary antibody overnight at 4°C and then with a horseradish peroxidase-conjugated secondary antibody for 1 h at room temperature. Bands were detected using enhanced chemiluminescence.

### Generation of knockout or knock-in A549 cells.

Knockout or knock-in A549 cells were generated using the CRISPR/Cas9 system as previously described (62). A lentiviral vector encoding EGFP-Cas9, the packaging plasmid pMD2.G, and the envelope plasmid psPAX2 were provided by Lisheng Zhang (63). The single-guide RNA sequence targeting the human GPS2 (5′-CTTGGGGCGCTCCAGGAGTG-3′) was cloned into the BsmBI site in the EGFP-Cas9 lentiviral vector and used to produce the recombined lentivirus. For GPS2-overexpressed A549 cells, the ORF of GPS2 was cloned into the BamHI/XbaI site in the pLenti-puro vector. Finally, the monoclonal cells acquired using the limiting dilution method were expanded, and the knockout of target genes was confirmed by Western blotting.
